# Norepinephrine Onset Time and Mortality in Patients with Septic Shock Treated in the Emergency Department

**DOI:** 10.3390/jcm14176025

**Published:** 2025-08-26

**Authors:** German Devia Jaramillo, Jose Wdroo Motta Hernández, William Gerardo Donoso Zapata

**Affiliations:** 1 Emergency Department, Hospital Universitario Fundación Santafé de Bogotá, Bogotá 111321, Colombia; 2 School of Medicine, Universidad del Rosario, Bogotá 111321, Colombia; josew.motta@urosario.edu.co (J.W.M.H.); william.donoso@urosario.edu.co (W.G.D.Z.)

**Keywords:** sepsis, septic shock, emergency department, mortality

## Abstract

**Introduction:** Sepsis, and particularly septic shock, is a life-threatening condition associated with high mortality rates in the emergency department. Timely interventions can significantly reduce these unacceptably high mortality rates. While some studies have demonstrated reduced mortality with early norepinephrine initiation, there is limited research on this intervention specifically within the emergency department setting. The objective of this study was to determine the association between the time to norepinephrine initiation in the emergency department and in-hospital mortality in adult patients diagnosed with septic shock. **Methods:** This retrospective cohort study included adult patients diagnosed with septic shock in the emergency department. Demographics, paraclinical variables, and the time to norepinephrine initiation were evaluated. In-hospital mortality was defined as the primary outcome. Finally, a multivariate analysis was performed to develop a nomogram for predicting septic shock mortality from the emergency department. **Results:** A total of 176 patients were included. A significant difference was documented between the time to norepinephrine initiation (in minutes) and survival rates: median (IQR) 12 (2–29) min for survivors versus 104 (68–181) min for non-survivors (*p* < 0.001). Similarly, when the time to initiation was divided into three groups (<60, 61–179, >179 min), a differential association with mortality was observed: OR 0.16 (95% CI; 0.08–0.32), OR 5.59 (95% CI; 2.67–11.6), and OR 353 (95% CI; 20.8–5978.9), respectively. Additionally, variables associated with mortality included mean arterial pressure, arterial lactate, and creatinine levels. **Conclusions:** Early initiation of norepinephrine in the emergency department may lower in-hospital mortality from septic shock without raising arrhythmia rates. Further high-quality studies are needed to confirm this and identify the patients who would benefit most.

## 1. Introduction

Septic shock stands as a leading cause of mortality in critically ill patients, particularly those managed in emergency departments, where timely and appropriate intervention is paramount [1]. This high mortality rate is particularly pronounced in low- and middle-income countries, such as those of Latin America [2]. In some low- and middle-income countries, sepsis mortality rates range from 19.4% to 21.8%, while those from septic shock are considerably higher, at 45.6% to 59.3% [3,4]. Even with ongoing therapeutic advancements in sepsis management, no universally applied treatment protocols have evidenced a substantial global reduction in sepsis mortality since the year 2001 [5]. Similarly, delays in the timely initiation of interventions for patients with septic shock increase the likelihood of unfavorable outcomes [6].

Emergency department overcrowding results in extended patient length of stay, a delay consistently associated with deteriorating clinical outcomes [7]. Given that sepsis is a time-sensitive disease requiring prompt interventions [8], it is a condition critically dependent on the timeliness of interventions; there is a need for strategies that are not merely opportune but also practically implementable within the emergency department. The emergency department often serves as the patient’s first contact. However, due to frequent overcrowding, interventions must be swift and easy to administer. Consequently, interventions demonstrating utility in the earliest stages of the disease are essential. Promptly implementing specific interventions, such as the early initiation of vasopressors in septic shock management, could substantially contribute to improved clinical outcomes [9]. There are several drugs used in patients with septic shock to improve blood pressure; some of these include vasopressin and analogues, norepinephrine, angiotensin II, and even methylene blue. Norepinephrine is an alpha1 and beta1 agonist and, therefore, can increase vascular tone and cardiac contractility [10]. It also increases cardiac preload and cardiac output in patients with life-threatening hypotension [9]. In this study, it was decided to use norepinephrine since it is the therapy that recent guidelines recommend as a first-line vasopressor in septic shock [10]. However, the key to vasopressor therapy may be the timing of drug initiation rather than the type of vasopressor chosen. A recent meta-analysis reported that the early initiation of norepinephrine in patients presenting with septic shock correlated with reduced short-term mortality, a quicker attainment of target mean arterial pressure, and a lower administered volume of intravenous fluids within the initial 6 h [10]. In contrast, a subsequent meta-analysis published in 2024, which compared early versus delayed norepinephrine administration in septic shock patients, reported no significant divergence in overall mortality [10]. Nevertheless, the early administration of norepinephrine seemed to mitigate the incidence of pulmonary edema. Furthermore, a discernible mortality improvement was noted in studies that did not implement fluid restriction interventions, thereby advocating for the early initiation of norepinephrine [11].

Ultimately, early norepinephrine initiation in septic patients could lead to improved clinical outcomes. However, many studies included in meta-analyses were conducted in intensive care unit (ICU) settings rather than emergency departments (EDs). Additionally, there is a lack of uniformity regarding the definition of “early initiation” of the medication. Therefore, the main objective of this study is to determine the association between the time to norepinephrine initiation in the emergency department and in-hospital mortality in adult patients diagnosed with septic shock, as well as to identify the optimal timeframe for the medication’s impact on mortality, explore whether other patient conditions are associated with this reduction in mortality, and evaluate the rate of arrhythmia triggered by the early initiation of the medication.

## 2. Materials and Methods

**Study Design:** This retrospective cohort study was designed to determine the association between the time to norepinephrine initiation in the emergency department and in-hospital mortality among adult patients diagnosed with septic shock. Data were collected from the sepsis database maintained by the Institute of Emergency and Trauma (ISMET) at Hospital Universitario Fundación Santa Fe in Bogotá, Colombia, between June 2023 and February 2025.

### 2.1. Eligibility Criteria

This study was conducted at a high-complexity university hospital in Bogotá, Colombia. We included adult patients aged 18 years or older who were evaluated in the emergency department (ED) with a diagnosis of septic shock, as defined by the Sepsis-3 consensus, irrespective of sepsis origin [12]. Patients transferred from other institutions, those previously receiving treatment at another center, and pregnant patients were excluded from this study. Furthermore, patients without confirmed infection were also excluded. All eligible patients were enrolled sequentially until the desired sample size was achieved.

### 2.2. Definition of Terms

**Infection Definition:** The diagnosis of infection was confirmed if the patient met at least one of the following criteria: (i) positive blood culture showing a non-colonizing, non-contaminating agent; (ii) positive urine culture (greater than 100,000 colony-forming units/mL), accompanied by urinary symptoms or sepsis without other apparent cause; (iii) positive culture from endobronchial aspirate, sputum, or bronchoalveolar lavage; (iv) positive ascitic fluid culture (greater than 250 polymorphonuclear cells per high-power field); (v) positive stool culture; or (vi) evidence of intra-abdominal collections or pulmonary consolidation in the absence of the findings above.

**Sepsis Definition:** Sepsis was diagnosed according to the current Sepsis-3 definition [12]. A case was classified as sepsis when infection was confirmed, and evidence of organ dysfunction was indicated by a Sequential Organ Failure Assessment (SOFA) score of 2 or more points.

**Septic Shock Definition:** Septic shock was defined per the Sepsis-3 definition as a subcategory of sepsis where profound circulatory, cellular, and metabolic abnormalities are associated with a greater mortality risk [13]. Its diagnostic criteria include hypotension, sustained vasopressor requirement to maintain a mean arterial pressure (MAP) ≥ 65 mmHg, and a serum lactate level greater than 2 mmol/L [12].

### 2.3. Sample Size Calculation

The sample size calculation was based on the anticipated incidence of the primary outcome: in-hospital mortality. Based on the previous literature, a mortality of 25% was estimated for early norepinephrine use versus 51.1% for late (6 h) use [14]. Considering an alpha level of 0.05 (corresponding to a critical Z-value of 1.96 for a 95% confidence level) and a power of 80% (corresponding to a crucial Z-value of 0.84 for an 80% power), the calculated sample size was 50.3 patients per group. Including a 10% allowance for potential attrition, 55.3 patients per group were required. This calculation was performed using R software, version 1.4.1106 (© 2009–2021 RStudio, PBC, Viena, Austria).

### 2.4. Error and Bias Control Strategies

- Information bias was controlled via systematic, orderly, and structured data collection by a dedicated research assistant using the prospective data collection instrument.
- Observer bias was controlled by direct data collection and variable values from medical records. The observer did not record any values, as this was a study in which no interventions were performed.
- Selection bias, despite the sample being from a single center, was controlled by the sepsis diagnostic criteria for study entry (sepsis-3). Furthermore, patient selection was controlled by the eligibility criteria used, and a representative sample of the population was ensured by calculating the sample size.

### 2.5. Data Analysis

The data recorded in the collection tool was meticulously reviewed to prevent inconsistencies or duplications, ensuring data integrity for each variable type. A descriptive analysis of the study variables was performed using frequency distributions for categorical variables. For continuous variables, measures of central tendency and dispersion (mean and standard deviation vs. median and interquartile range (IQR) for standard and non-normal distributions, respectively) were calculated, applying the Shapiro–Wilk test for normality assessment.

Bivariate analyses were conducted to explore associations with the mortality outcome. For categorical variables, differences in mortality were determined using the Chi-squared or Fisher’s exact test, depending on the data distribution. For continuous variables, differences were assessed using a Student’s *t*-test or the Mann–Whitney U test, based on the normality of the data distribution. Finally, a multivariate analysis was proposed using a logistic regression model to estimate odds ratios for the risk of in-hospital death. For the analysis, we used two types of strategies: forward and backward, according to the statistical significance of each variable with the outcome variable “mortality”. The variables included in the model were age, sex, site of infection, time of norepinephrine initiation, PAFI value, platelets, mean arterial pressure, Glasgow Coma (GCS), bilirubin, creatinine, lactate, and SOFA score. The models found using the different methods were then compared through nested model analysis using the likelihood ratio test, as well as through analysis of the AIC (AKAIKE) value and the adjusted R^2^ (coefficient of determination). After comparing the models, the best model was selected. An ROC curve was constructed to discriminate the model. Finally, an internal validation method was performed using a bootstrap simulation with 1000 repetitions. A *p*-value of less than 0.05 was considered statistically significant. All statistical computations were performed using RStudio software, version 2024.12.1+563, Copyright (C) 2025 by Posit.

## 3. Results

**Figure 1:**
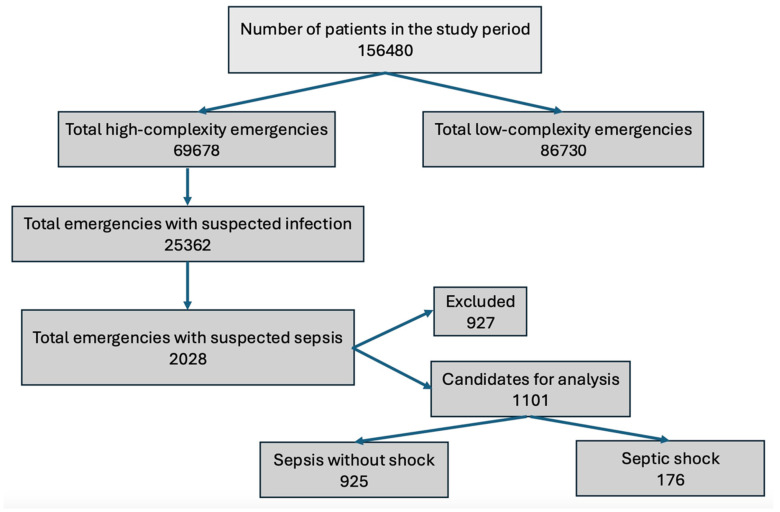
Flowchart for patient inclusion.

Of the eligible patient population, 176 patients were included in the final analysis (Figure 1). The overall in-hospital mortality for the cohort was 31.8%. No significant difference in mortality was observed by sex or age. The most frequent sites of infection were urinary (31.2%) and pulmonary (17.6%) (Table 1). Generally, mortality did not differ significantly across infection sites, except for pulmonary focus, which showed a statistically significant association with mortality [odds ratio (OR), 2.73 (95% CI, 1.35–5.51)] (Table 1).

Regarding laboratory variables, lactate and creatinine levels and the total Sequential Organ Failure Assessment (SOFA) score were significantly associated with mortality (Table 1). Furthermore, the only comorbidity significantly associated with mortality was a history of oncological disease (Table 1). Interestingly, arrhythmias were not significantly associated with mortality (Table 1).

**Figure 2:**
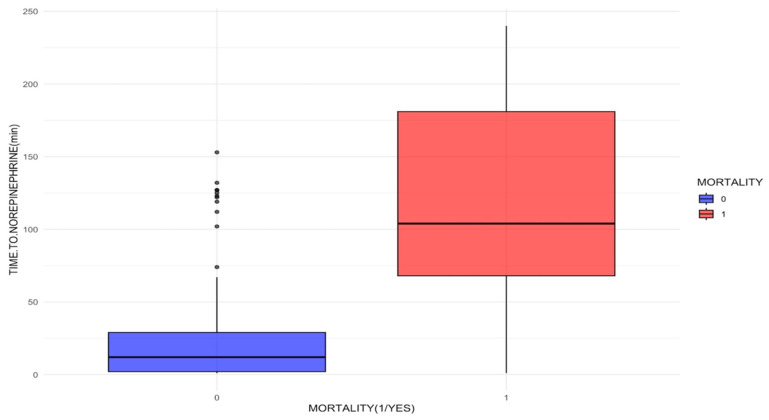
Mortality and time to norepinephrine onset in ED.

Concerning the association between the time to norepinephrine initiation and mortality, a significant difference was documented in the median time to medication initiation (in minutes) between survivors and non-survivors (Figure 2). The median time to norepinephrine initiation was 12 minutes (interquartile range [IQR], 2–29) for survivors vs. 104 min (IQR, 68–181) for non-survivors (Table 2). Specifically, initiation of norepinephrine within less than 60 min was associated with an OR of 0.16 (95% CI, 0.08–0.32), whereas initiation of the medication after 180 min was associated with an OR of 353 (95% CI, 20.8–5978.9) (Table 2).

**Figure 3:**
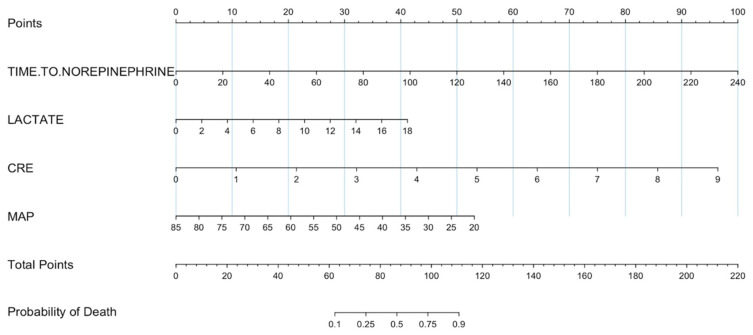
Death prediction nomogram. CRE: Creatinine; MAP: mean arterial pressure.

When performing the logistic regression analyses, we documented two models with a good fit. The difference between the two models was the variable site of infection. However, when comparing the models, we determined that the model that did not include the site of infection was more parsimonious than the larger model, maintaining good performance, so the model with fewer variables was left in. The variables that remained in the model were the time until the start of treatment with norepinephrine (in minutes), the lactate level upon admission to the emergency department, the creatinine level, and the mean arterial pressure on admission. This model demonstrated a high discriminatory capacity with an area under the receiver operating characteristic curve (AUC) of 0.934 (95% CI, 0.892–0.964) (Figure S1). Based on this developed model, a nomogram was created to predict in-hospital mortality from septic shock diagnosed in the emergency department (Figure 3).

## 4. Discussion

This study determined that the time to norepinephrine initiation was significantly associated with observed in-hospital mortality. Efforts to optimize vasopressor management should be included in the ED. Determining the optimal vasopressor approach for managing the different phases of septic shock is a current priority in clinical research [15]. This distinction is crucial due to the unique characteristics of ED care, primarily driven by the time constraints imposed by persistent overcrowding [16].

Initiation within less than 60 min was linked to a significant decrease in mortality [OR, 0.16 (95% CI, 0.08–0.32)]. Conversely, for patients whose medication was initiated between 61 and 179 min, mortality significantly increased [OR, 5.59 (95% CI, 2.67–11.6)]. Furthermore, all patients in the cohort who received norepinephrine after 180 min ultimately died. This underscores the critical role of timely intervention in septic shock, a complex pathophysiological state characterized by vasodilation due to decreased vascular tone, relative and absolute hypovolemia, myocardial dysfunction, increased metabolic rate, and impaired regional and microvascular blood flow, with hypotension being a key clinical correlate [17,18,19]. Therefore, the management of septic shock necessitates a medication capable of increasing vascular tone and, to some extent, myocardial contractility. This explains why norepinephrine, acting as an alpha-1 and beta-1 agonist, is included in recent treatment guidelines as the first-line vasopressor for septic shock due to its ability to enhance vascular tone and contractility [20].

Multiple studies have focused on the rational use of various vasopressor types [21,22,23]. However, given the less robust results of these studies, it appears that not only the agent used for shock management but also the timing of its initiation should be considered. This aligns with the findings documented in our study and the meta-analysis by Li Y et al. [10], which demonstrated that early norepinephrine initiation in septic shock patients was associated with lower short-term mortality [OR, 0.45 (95% CI, 0.34–0.61)], a shorter time to reach target mean arterial pressure [mean difference, −1.39 (95% CI, −1.81 to −0.96)], and a lower volume of intravenous fluids within 6 h [mean difference, −0.50 (95% CI, −0.38 to −0.62)]. A key limitation of this meta-analysis, which included only five studies, was its mixed patient population, encompassing both intensive care unit (ICU) and emergency department (ED) settings. Furthermore, the definition of “early initiation” was not uniform across the analyzed studies, varying widely from 1 h to 2 h, 93 min, and even up to 6 h, which significantly complicated the interpretation of their results. Given these limitations, the meta-analysis by Ahn C et al. [11], which included 12 studies (4 randomized controlled trials), found no significant difference in overall mortality between early vs. late norepinephrine initiation groups, whether in randomized controlled trials [OR, 0.70 (95% CI, 0.41–1.19)] or in observational studies [OR, 0.83 (95% CI, 0.54–1.29). Another meta-analysis [24], which compared 10 studies (including only 2 randomized clinical trials), documented a reduction in mortality with early norepinephrine administration in both randomized clinical trials [OR, 0.49 (95% CI, 0.25–0.96)] and observational studies [OR, 0.71 (95% CI, 0.54–0.94)]. However, the sequential trial analysis for mortality revealed that the cumulative Z-curve neither crossed the futility boundary nor reached the required information size, suggesting insufficient evidence and a non-conclusive result.

The limitations of this meta-analysis include a heterogeneous study setting, with three studies conducted in the emergency department, one in a prehospital setting, and one in a mixed environment, while five studies were performed in intensive care unit populations. Additionally, it also lacked uniformity in the definition of early norepinephrine initiation.

We compare the results of our study with research conducted in an emergency department setting. For instance, in a randomized clinical trial by Permpikul et al. [9], norepinephrine initiation was compared with a placebo for septic shock treatment in the emergency department (ED). The authors found that the median time from ED arrival to norepinephrine administration was significantly shorter in the early norepinephrine group (93 vs. 192 min; *p* < 0.001). Furthermore, the 6 h shock control rate was significantly higher in the early norepinephrine group (76.1% vs. 48.4%; *p* < 0.001). However, 28-day mortality did not show a difference between the groups (15.5% vs. 21.9%; *p* = 0.15). Similarly, a study involving 101 emergency department patients compared the simultaneous initiation of norepinephrine and fluids versus norepinephrine initiation after fluid failure [25]. This study documented that the early norepinephrine group initiated infusion at a median of 25 min (95% CI, 20–30 min) compared to 120 min (95% CI, 120–180 min) for the late group. A more rapid and significant improvement in mean arterial pressure was achieved with early norepinephrine initiation. Norepinephrine commenced at a median of 30 min (IQR, 20–120 min) in survivors versus 120 min (IQR, 30–165 min) in non-survivors (*p* = 0.013). These data are like the findings in the current study, where norepinephrine was initiated at a median of 12 min (IQR, 2–29 min) in survivors, compared to 104 min (IQR, 68–181 min) in non-survivors (*p* < 0.001). Based on the mean minutes of norepinephrine onset found in the survivor group in our study, as well as the mean reported in the studies performed in the emergency department setting by Permpikul et al. [9] and Elbouhy MA et al. [25], we consider the most appropriate cut-off point for considering “early onset” to be the first 60 min of a patient’s stay in the emergency department with septic shock. We believe it should be emphasized that it is not about the start of vasopressor medication, but rather the improvement in mean arterial pressure in the first 60 min.

In contrast to these studies, the work by Yeo et al. [26], involving 298 retrospectively collected patients, documented that norepinephrine initiation within less than 60 min was associated with an increase in in-hospital mortality (51.7% vs. 39.6%; *p* = 0.036). This study, however, had inherent limitations common to retrospective designs, notably heterogeneity in management protocols across participating institutions. This institutional variation likely introduced confounding factors, making it challenging to definitively establish the association between mortality and early norepinephrine use.

Generally, we contend that the critical factor is not solely the early administration of norepinephrine, but rather the opportune timing of its initiation, alongside individualized fluid management. It is conceivable that certain patients may benefit from the simultaneous early administration of both interventions. Indeed, a study conducted in a population like ours, which employed individualized fluid and vasopressor administration guided by ultrasound, demonstrated that this personalized approach led to a significantly faster recovery of mean arterial pressure. Additionally, fluid balances were lower in the intervention group compared to the control group, with a discernible trend towards reduced mortality [27]. No significant difference in the incidence of arrhythmias was documented between the groups, a finding similar to that reported in the study by Yeo et al. [26]. This suggests that norepinephrine administration is safe.

The cohort’s overall in-hospital mortality rate was 31.8%. This finding is comparable to the 34.7% septic shock mortality rate reported by Bauer et al. [28] and the 26% rate in the study by Fleischmann et al. [29]. Furthermore, the septic shock mortality rate observed in our cohort was similar to that reported in other low-to-middle-income populations (34.9%) [30].

No significant difference in cohort mortality was documented based on the site of infection, except for the pulmonary focus. For other infection sites where differences appeared, statistical values indicated an insufficient number of cases to be considered significant. Similarly, no difference in mortality was found across comorbidities, except in patients with a history of oncological disease. This finding aligns with previous reports indicating that sepsis mortality in hospitalized cancer patients can reach 37.8% versus 24.9% in non-cancer patients [31].

Finally, a nomogram for predicting septic shock mortality from the emergency department was developed based on logistic regression. This nomogram incorporates variables such as lactate value, creatinine level, and mean arterial pressure at admission. These data, combined with the time of norepinephrine initiation, can assist clinicians in estimating the probability of death for treated patients and inform decisions regarding more aggressive and timely interventions. While this nomogram did not include important variables such as the volume of fluids administered or the time to antibiotic initiation, which could potentially enhance the discriminatory capacity of the tool, we documented an adequate predictive capability with an area under the receiver operating characteristic curve (AUC) of 0.934 (95% CI, 0.892–0.964). Based on the information documented in this study, it is likely that the patients who would benefit most from considering starting norepinephrine within 60 min (early start) are the patients who present to the emergency department with low mean arterial pressure associated with elevated arterial lactate and elevated creatinine.

Achieving the goal of early initiation of vasopressor support in the emergency department is not easy and represents a major challenge. However, the emergency physician must identify early which patients would benefit from early initiation of vasopressor therapy, in addition to fluid therapy, to improve blood pressure, since this type of intervention in the emergency department will influence the prognosis of patients with septic shock. Studies such as the one contained in this manuscript can be used as useful tools for clinicians to improve patient outcomes.

### 4.1. Limitation

The primary limitation of this study is its single-institution design, which suggests that the results should be interpreted cautiously when generalizing to other populations. However, the patient mortality rates observed were comparable to those reported in previously studied cohorts. Additionally, this study utilized retrospective data, which carries the risk of collection biases. Nevertheless, we double-verified the data to mitigate inconsistencies in the entered results. The wide confidence intervals in some results could be attributed to the sample size within their subgroups. While a formal sample size calculation was performed for the overall study, these variations are expected given the study design and are not believed to affect the primary findings significantly. Although our study’s results are comparable to those obtained in some meta-analyses, these findings do not warrant the systematic, routine use of early norepinephrine. In addition to their possible relationship with arrhythmias, premature initiation of vasopressors could underestimate the hemodynamic status and limit fluid resuscitation, which theoretically could worsen tissue perfusion under conditions of absolute or relative hypovolemia [26]. We did not continuously evaluate lactate clearance in the groups or the need for renal replacement therapy; we consider this a possible limitation of this study. However, it is important to clarify that the characteristics of the groups were comparable and that a significant decrease in mortality was demonstrated. Despite this, we believe it is prudent to design new studies to evaluate these variables over time. We advocate that early norepinephrine should be considered for all septic shock patients managed in the emergency department. Still, an individualized approach is essential, given the existing literature on the potential harm of this intervention.

### 4.2. Perspectives for the Future

The findings of this study support the consideration of the early concomitant use of fluids and norepinephrine in patients with septic shock identified in the emergency department instead of fluids alone, especially in patients with elevated lactate and creatinine levels.

It was demonstrated that improving mean arterial pressure within the first hour after identifying a patient with septic shock should be a priority in the care of patients in the emergency department.

However, further studies are needed to evaluate the effects of early norepinephrine therapy and lactate clearance over time as an indicator of cellular perfusion, as well as the need for renal replacement therapy, hematologic alterations such as hemodilution, interstitial edema, and microcirculatory abnormalities.

## 5. Conclusions

In conclusion, based on the results of this study, early norepinephrine initiation in the emergency department may decrease in-hospital mortality from septic shock without increasing arrhythmia rates. These findings suggest that this intervention is reasonable. However, high-quality studies are needed to routinely recommend this intervention and, more specifically, to determine which patient population will benefit most.

## Figures and Tables

**Table 1:** Characteristics of the patients.

Variable	Survivors (%)	No Survivors (%)	OR	*p*
**Total (n = 176)**	120 (68.2)	56 (31.8)		
**Sex (male)**	60 (50.0)	21 (37.5)	0.60 (0.31–1.14)	0.165
**Age (median (IQ))**	74 (63.5–81.5)	74.5 (68–86.2)		0.663
**Sepsis origin (%)**				0.087
**Urinary**	47 (39.2)	12 (21.4)	0.76 (0.37–1.53)	0.445
**Pulmonary**	20 (16.7)	18 (32.1)	2.73 (1.35–5.51)	0.005
**Gastrointestinal**	21 (17.5)	12 (21.4)	3.04 (1.36–6.78)	0.006
**Biliary tract**	10 (8.3)	2 (3.6)	2.93 (0.57–14.92)	0.194
**Soft tissues**	6 (5)	4 (7.1)	11.73 (2.84–48.37)	<0.001
**Abdominal**	6 (5)	6 (10.7)	14.66 (4.10–52–42)	<0.001
**Bacteremia without focus**	6 (5)	0 (0)	2.08 (0.10–41.16)	0.628
**Indeterminate**	2 (1.7)	1 (1.8)	29.33 (2.05–418.50)	0.012
**Endocarditis**	1 (0.8)	1 (1.8)	88 (3.95–1958.41)	0.004
**Malaria**	1 (0.8)	0 (0)	39.22 (1.09–1409.1)	0.044
**Glasgow score (median (IQ))**	15 (14–15)	15 (14.75–15)		0.891
**Systolic blood pressure (mean (SD))**	74.9 (10.8)	72.6 (15.5)		0.265
**Diastolic blood pressure (median (IQ))**	45 (40–49.2)	42 (38–50)		0.134
**Mean arterial pressure (median (IQ))**	56 (51–61)	54.5 (46.7–60)		0.078
**PaFi (median (IQ))**	253.5 (205.8–301.2)	233 (122.2–292.2)		0.058
**Platelet count (median (IQ))**	194.5 (138–278)	216 (129–306.8)		0.700
**Bilirubin (median (IQ))**	1.1 (0.7–1.9)	1.2 (0.6–1.8)		0.544
**Creatinine (median (IQ))**	1.2 (0.9–1.7)	2.1 (1.4–2.9)		<0.001
**Lactate levels (median (IQ))**	2 (1.2–3.5)	6 (2.5–8.2)		<0.001
**SOFA (median (IQ))**	5 (4–6)	6 (5–8)		<0.001
**Comorbidities**				
**Immune diseases**	13 (10.8)	7 (12.5)	1.18 (0.41–3.11)	0.945
**Cardiovascular diseases**	60 (50)	30 (53.6)	1.15 (0.60–2.19)	0.779
**Endocrinological diseases**	41 (34.2)	24 (42.9)	1.44 (0.74–2.77)	0.344
**Liver diseases**	17 (14.2)	8 (14.3)	1.01 (0.38–2.48)	1.000
**Chronic infections**	2 (1.7)	2 (3.6)	2.17 (0.22–21.34)	0.805
**Neurological diseases**	8 (6.7)	3 (5.4)	0.81 (0.16–3.02)	1.00
**Oncological diseases**	27 (22.5)	23 (41.1)	2.38 (1.19–4.76)	0.018
**Kidney failure**	17 (14.2)	8 (14.3)	1.01 (0.38–2.48)	1.00
**Lung diseases**	12 (10)	9 (16.1)	1.72 (0.65–4.39)	0.364
**Presence of arrhythmias**	5 (4.2)	6 (10.7)	2.72 (0.76–10.1)	0.181
**Time stay outside ICU (median (IQ))**	12 (9–18)	4.5 (3–7.7)		<0.001
**Stay in ICU (median (IQ))**	22 (13.7–24)	17.5 (1–22.5)		<0.001

PaFi, arterial oxygen pressure divided by the fraction of inspired oxygen; SOFA, Sequential Organ Failure Assessment; Time stay outside ICU: Total in-hospital stay minus total ICU stay.

**Table 2:** Norepinephrine onset time and mortality.

Variable	Survivors	No Survivors	OR	*p*
Norepinephrine onset time (median (IQ)) min	12 (2–29)	104 (68–181)		<0.001
Time to norepinephrine (%)				
60 min or less	103 (85.8)	11 (19.6)	5.59 (2.67–11.6)	<0.001
61 to 179 min	17 (14.2)	20 (35.7)	5.59 (2.67–11.6)	<0.001
180 min or more	0 (0.0)	25 (44.6)	353 (20.8–5978.9)	<0.001

## Data Availability

The datasets used and/or analyzed in the current study are available from the corresponding author upon reasonable request.

## Supplementary Materials

The following supporting information can be downloaded at https://www.mdpi.com/article/10.3390/jcm14176025/s1, Figure S1: Receiver operating characteristic curve of the created model.

## Abbreviations

AUC	Receiver operating characteristic curve
PaFi	Arterial oxygen pressure divided by the fraction of inspired oxygen
SOFA	Sequential Organ Failure Assessment
ICU	Intensive care unit
HM	Hedonic motivation
Min	Minutes
MAP	Mean arterial pressure
