# Supplementary figures and images for: Norepinephrine Onset Time and Mortality in Patients with Septic Shock Treated in the Emergency Department
Source: J Clin Med. 2025 Aug 26;14(17):6025. doi: 10.3390/jcm14176025 (PMC12429137; doi:10.3390/jcm14176025)

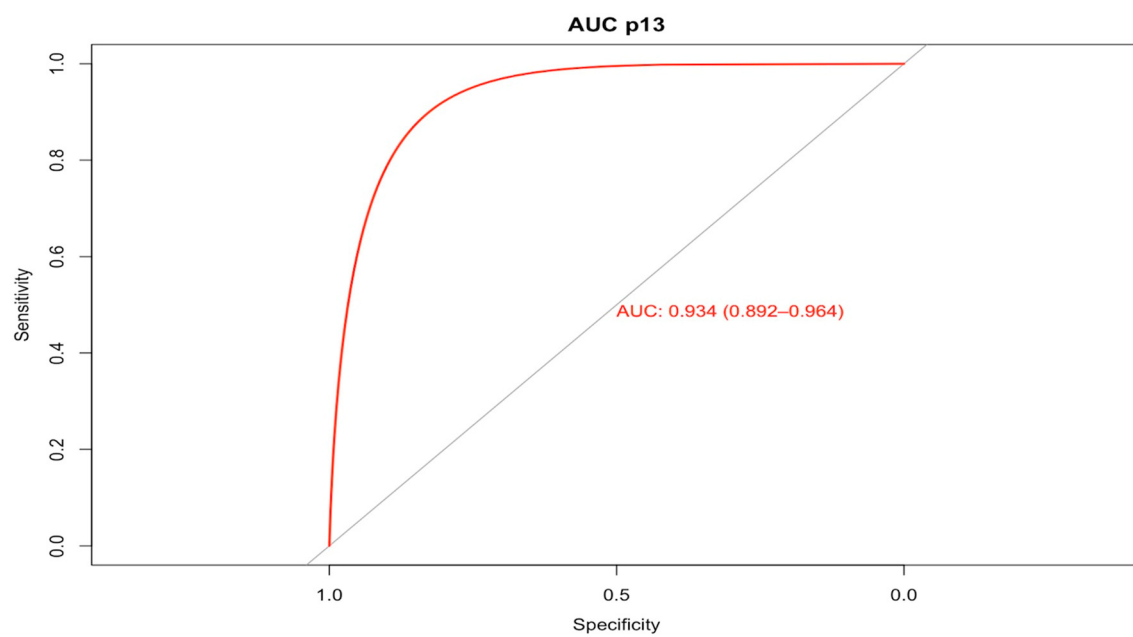

**Figure S1.** Receiver operating characteristic curve of the created model.

Supplement: Supplementary file 1 [file jcm-14-06025-s001.zip › jcm-3830177-supplementary.pdf]
